# Worst Oxygenation Index during the first 24 hours of ventilation predicts mortality

**DOI:** 10.1186/cc10697

**Published:** 2012-03-20

**Authors:** RJ Jackson, TH Gould, MJ Thomas

**Affiliations:** 1 Bristol Royal Infirmary, Bristol, UK

## Introduction

The ratio of PaO_2 _to FiO_2 _(P/F ratio) is often used to classify patients with hypoxic respiratory failure, and is recommended in guidelines from a UK expert group [1] but does not take airway pressures into account. A study found that adjusting for PEEP did not affect the predictive ability of the P/F ratio [2]; however, the mean airway pressure (MAP) may be a better indicator of lung recruitment. The Oxygenation Index (OI = (FiO_2_×MAP)/PaO_2_)) includes an adjustment for MAP.

## Methods

We retrospectively assessed a computerised record (from 2008 to 2010) of ventilator parameters and identified the highest OI for all ventilated patients from a general adult university teaching hospital ICU, during the first 24 hours of ventilation. Patients were grouped according to highest OI, and mortality was calculated for subgroups.

## Results

**Figure 1:**
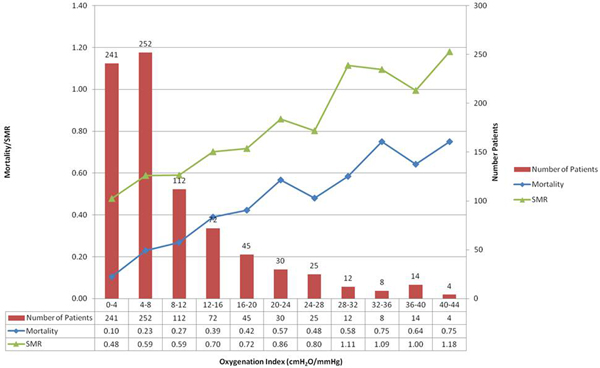
**Mortality and number of patients by Oxygenation Index**.

Data were available for 815 patients (see Figure 1). Increasing OI was associated with increasing mortality (*P *< 0.0001 chi-squared test for trend). Each step increase in OI was associated with approximately a 6% absolute increase in mortality. The OI was also associated with increasing Standardised Mortality Ratio (ICNARC model).

## Conclusion

The highest OI occurring in the first 24 hours of ventilation is an independent predictor of mortality. Collection of OI data may allow better prediction of outcome than P/F ratio data alone.
